# Artificial intelligence technologies in pregnancy and childbirth care: a scoping review from a nursing perspective

**DOI:** 10.4069/whn.2026.09.04

**Published:** 2026-09-29

**Authors:** Hyunjin Cho, Sukhee Ahn

**Affiliations:** 1 College of Nursing, Chodang University, Muan, Korea; 2 College of Nursing, Chungnam National University, Daejeon, Korea

**Keywords:** Artificial intelligence, Machine learning, Nursing practice, Pregnancy, Pregnancy complications

## Abstract

**Purpose:**

This scoping review examined the applications of artificial intelligence (AI) technologies in pregnancy and childbirth care, including patterns of use and outcomes relevant to nursing practice.

**Methods:**

Using Arksey and O’Malley’s methodological framework, we comprehensively searched PubMed, CINAHL, Embase, and Web of Science for empirical studies published from 2015 to 2025. Of the 1,282 records identified, 1,191 were screened after duplicates were removed, and 20 studies of pregnancy and childbirth care were included.

**Results:**

AI applications in the 20 included studies were grouped into four domains: risk prediction models (n=14), fetal/intrapartum monitoring (n=3), clinical decision support (n=1), and patient education and nursing documentation (n=2). Publications increased markedly from 2021 onward. Machine learning algorithms, including random forest, support vector machine, extreme gradient boosting, and logistic regression, were used mainly to predict obstetric complications such as preterm birth, hypertensive disorders of pregnancy, preeclampsia, and gestational diabetes mellitus. AI models also analyzed fetal heart rate and electrocardiogram signals to support intrapartum decisions, including prediction of episiotomy risk and vaginal birth after cesarean delivery. A robot-assisted education system supported patient education, and a large language model supported nursing documentation.

**Conclusion:**

AI is used primarily for risk prediction and clinical decision support in pregnancy and childbirth care, with emerging applications in patient education and psychosocial assessment. Evidence for nurse-led AI interventions is limited. Research should prioritize clinical validation, AI literacy education, nurse-led program development, and ethical guidelines to support safe, equitable, individualized perinatal nursing care.

## Introduction

Artificial intelligence (AI), a central technology of the 21st-century Fourth Industrial Revolution, is being adopted rapidly in medicine to improve diagnostic accuracy, personalize treatment, and support prediction-based health management [1]. As demand for digital healthcare has increased since the coronavirus disease 2019 pandemic, AI has been integrated with remote monitoring, mobile health applications, chatbot counseling, and analysis of biosignals from wearable devices, changing the delivery of healthcare services [2]. These developments also offer new possibilities for health management during pregnancy, the most sensitive and critical period in a woman’s life.

Pregnancy involves pronounced physical and psychological changes that profoundly affect maternal and fetal health and the quality of life of the entire family [3]. Some pregnant women experience high-risk conditions, including hypertensive disorders of pregnancy, preeclampsia, gestational diabetes mellitus (GDM), and antenatal/perinatal depression; these complications are closely associated with increased maternal mortality [4].

Conventional prenatal care assesses maternal health through vital signs, blood tests, and fetal ultrasonography at regular outpatient visits. Electronic medical records are used to compare and analyze changes in maternal and fetal health [5]. Digital healthcare extends beyond these visits by allowing real-time collection and analysis of maternal health data through wearable devices, remote monitoring, and AI-based prediction tools [6,7]. This change requires adjustments to patient assessment and intervention timing, including opportunities for earlier intervention.

Efforts are therefore expanding to use AI for more timely monitoring of maternal health, early detection of potential risk factors, and systematic, individualized maternal health management [6]. Maternal nursing combines holistic, person-centered care with evidence-based practice [8] and emphasizes nurses’ distinctive roles in emotional support, health education, and continuous health management [9]. AI applications in pregnancy nursing could change both the nature of care and clinical practice [6,7], but few studies have comprehensively examined these applications and their implications from a nursing perspective. A systematic examination of AI use in maternal health management is therefore needed to clarify its relevance to nursing practice as digital healthcare changes.

Previous studies have reported benefits of AI in nursing education and practice, including greater work efficiency, support for clinical decisions, and improved patient safety [9-12]. Recent work has integrated AI with wearable devices and the Internet of Things to monitor maternal and fetal biosignals continuously [13]. Real-time alerts and remote management are intended to address gaps in prenatal care, particularly in areas with limited healthcare access [14-16]. However, AI research in nursing often addresses only specific clinical situations or aspects of practice, or remains preliminary without links to real-world clinical data and scenarios [10,15,17].

Studies of AI applications in high-risk pregnancies involving GDM, hypertensive disorders of pregnancy, or preterm birth are increasing, but most emphasize technical outcomes such as prediction accuracy, comparative algorithm performance, and model efficiency [5]. Systematic reviews that comprehensively examine AI use in maternal health management remain at an early stage. Few studies discuss the outcomes of clinical decision support systems in maternal health management or their relationship to nursing practice. Research is particularly limited on the scope and characteristics of AI-based nursing interventions, including midwifery practice; nurses’ roles and competencies when using AI; and the limitations and potential risks of these technologies. Discussion within nursing and empirical evidence on integrating AI into maternal nursing practice, including early identification of high-risk pregnancies and preventive interventions, also remain limited [10,14,18].

This study therefore aimed to examine research on AI applications in maternal health management and systematically analyze the purposes and methods of those applications. By reviewing the evidence from a nursing perspective, it also sought to characterize AI use in maternal nursing practice and provide a basis for integrating AI effectively into future pregnancy nursing practice and research.

This scoping review analyzed AI technologies used in maternal health management and explored their potential links to nursing practice. Its specific objectives were as follows:

(1) To identify the types and characteristics of AI technologies used for risk prediction, fetal monitoring, and remote monitoring in maternal health management.

(2) To identify the types and characteristics of AI technologies used in educational and chatbot applications for maternal health management.

(3) To explore how AI applications in maternal health management relate to nursing practice and identify their implications for nursing.

## Methods

**Ethics statement:** This review examined existing literature and involved no human participants. Institutional review board approval and informed consent were therefore not required.

### Study design

This scoping review followed the methodological framework of Arksey and O’Malley [19] and the recommendations of Peters et al. [20]. It comprised five stages: (1) identifying the research question, (2) identifying relevant studies, (3) selecting studies, (4) charting the data, and (5) collating, summarizing, and reporting the results. The review followed the scoping review methodology recommended by the Joanna Briggs Institute (JBI) and was reported according to the Preferred Reporting Items for Systematic Reviews and Meta-Analyses extension for Scoping Reviews (PRISMA-ScR) guidelines [21]. To promote transparency, the review was prospectively registered in PROSPERO (CRD420251044613) before data collection.

### Research question

The primary research question was: How is AI used in prenatal and intrapartum health management, and what are the current patterns of use and outcomes from a nursing practice perspective?

### Search strategy

#### Stage 1. Identifying the research question

This scoping review addressed the following question:

How is AI used in prenatal and intrapartum health management, and what are the current patterns of use and outcomes from a nursing practice perspective?

The research question was developed using the population, concept, and context framework recommended by the JBI. The population comprised women who had experienced pregnancy. The concept was the application of AI technologies, including machine learning (ML), deep learning, natural language processing, and chatbots, to maternal health management, together with related outcomes such as risk prediction, diagnostic support, and health promotion. The context comprised the outcomes of risk prediction, diagnosis, and health promotion in relation to maternal health.

#### Stage 2. Identifying relevant studies

Four major healthcare and nursing databases were searched for studies of AI in maternal health management: PubMed/MEDLINE, the CINAHL, Embase, and Web of Science. The search covered publications from January 2015 to December 2025 to capture research trends beginning in the mid-2010s, when deep learning-based AI began to be used in medicine for clinical decision support and predictive model development [22]. Eligible publication languages were English and Korean.

The search combined AI terms (“artificial intelligence,” “machine learning,” “deep learning,” “neural network,” “predictive model,” “natural language processing,” and “chatbot”), pregnancy terms (“pregnancy,” “prenatal,” “antenatal,” “perinatal,” “gestational,” “obstetric,” “labor,” and “postpartum”), and nursing terms (“nursing” and “midwifery”). Medical Subject Headings and text words were adapted to each database (Supplementary Material 1).

#### Stage 3. Study selection

The inclusion criteria were: (1) studies involving women who had experienced pregnancy; (2) studies applying AI technologies, such as ML, deep learning, and natural language processing, to maternal health management, including prenatal and intrapartum care, and reporting related outcomes; (3) studies addressing nursing or midwifery practice or with applications to these practices in clinical settings; and (4) peer-reviewed articles published in English or Korean.

Studies were excluded if they (1) did not report empirical findings, including narrative literature reviews, opinion pieces, and commentaries; (2) reported only a study protocol; or (3) focused exclusively on the postpartum period.

Two researchers independently selected the studies. After removing duplicate records from the database searches, they screened titles and abstracts and excluded studies that did not meet the inclusion criteria. The remaining articles were assessed in full to determine final eligibility. Uncertainty about inclusion was resolved through discussion until the researchers reached consensus. The PRISMA-ScR checklist was used to support transparent reporting of study selection.

#### Stage 4. Charting the data

Data from the included studies were charted using an extraction form developed in advance by the researchers in Microsoft Excel (Microsoft Corporation, Redmond, WA, USA). The form captured authors, publication year, country, study design, study population, AI technology, application domain in maternal health management (risk prediction, fetal monitoring, remote monitoring, education, and chatbots), main findings, and links to nursing practice. Two researchers extracted the data independently. When information was unclear, they jointly re-examined the study and reached consensus. Discrepancies in extraction were also resolved by agreement between the two researchers.

#### Stage 5. Collating, summarizing, and reporting the results

The searches identified 1,282 records: 567 from PubMed, 51 from CINAHL, 370 from Web of Science, and 294 from Embase. After 91 duplicates were removed, the titles and abstracts of 1,191 records were screened, and 1,101 studies unrelated to the review topic were excluded. The remaining 90 studies underwent full-text review. Of these, 48 were excluded because they did not involve pregnant women (n=35), did not use AI (n=5), reported outcomes unrelated to the review purpose (n=4), or provided insufficient information for data extraction, such as AI methods, outcome indicators, or participant characteristics (n=4). This left 42 initially included studies. A further 22 studies focused on postpartum health management and were excluded, leaving 20 studies of maternal health management for analysis. Figure 1 presents the study selection process in a PRISMA-ScR flow diagram.

Critical appraisal was not conducted, consistent with the scoping review approach described by Arksey and O’Malley [19] and Peters et al. [20]. This approach maps the scope, extent, and characteristics of existing research rather than evaluating its methodological quality. The 20 included studies used diverse designs, including retrospective cohort studies, a randomized controlled trial (RCT), model development and validation studies, and retrospective signal analyses.

Two researchers independently extracted and cross-checked the data before organizing and summarizing them. Descriptive statistics summarized the general study characteristics, while thematic organization described AI application domains and their links to nursing practice. The synthesis and presentation were reviewed iteratively to maintain alignment with the research question and accurately represent the included studies.

## Results

### Characteristics of included studies

The review included 20 studies of AI in maternal health management (Table 1). Few were published in the earlier years: one (5%) in 2017, two (10%) in 2021, and one (5%) in 2022. Publication increased to nine studies (45%) in 2024 and seven (35%) in 2025; together, 2024 and 2025 accounted for 80% of the included studies.

The studies involved nine countries. China accounted for seven studies (35%) and Taiwan for three (15%). Türkiye and the United States each contributed two studies (10%), while Brazil, Ethiopia, the United Kingdom, Greece, and Australia each contributed one (5%). One additional multinational study (5%) involved China and the United Kingdom. Research was concentrated in Asia, particularly China and Taiwan in East Asia.

Retrospective cohort studies predominated, accounting for 14 studies (70%). The other designs comprised two retrospective signal analyses (10%), one population-based study (5%), one model development and validation study (5%), one RCT (5%), and one quality improvement study (5%). Most studies were retrospective; prospective and interventional designs were uncommon, with one study each.

ML-based prediction models were the most common AI technology, used in 15 studies (75%). The main algorithms were random forest, support vector machine (SVM), extreme gradient boosting (XGBoost), and logistic regression, primarily used to predict pregnancy complications and classify risk factors. Two studies (10%) combined biosignal analysis with ML: one classified delivery mode using fetal heart rate (FHR) signals [23], and the other monitored labor pain using electrocardiogram (ECG) waveforms [24]. The fuzzy analytic hierarchy process (FAHP) was used to develop a preterm birth risk assessment model incorporating socioeconomic, behavioral, and medical factors [25]. A robot-assisted hybrid system delivered digital education to improve maternal health literacy [26]. A study of ChatGPT-4, a large language model (LLM) of OpenAI (San Francisco, CA, USA) [27], examined its use in documenting pregnancy nursing care plans. This direct application of an AI documentation tool to nursing practice explored whether it could improve care plan quality and reduce documentation burden.

Risk prediction was the most common application domain, represented in 14 studies (70%), followed by fetal/intrapartum monitoring in three (15%). Clinical decision support, patient education, and nursing documentation each accounted for one study (5%).

By pregnancy stage, 15 studies (75%) addressed the prenatal period, three (15%) the intrapartum period, and two (10%) the first trimester. Preterm birth was the most frequently studied health outcome (four studies, 20%), followed by GDM (three, 15%). Antenatal/perinatal depression and preeclampsia were each examined in two studies (10%). General high-risk pregnancy, severe maternal morbidity (SMM), episiotomy, delivery mode/vaginal birth after cesarean delivery (VBAC), cesarean delivery classification, labor pain assessment, low birth weight, maternal health literacy, and nursing care plan documentation were each addressed in one study (5%).

### Patterns of use by artificial intelligence application domain

The 20 studies were grouped into four AI application domains: risk prediction models (n=14), fetal/intrapartum monitoring (n=3), clinical decision support (n=1), and patient education and nursing documentation (n=2) (Table 2, Supplementary Material 2). Risk prediction accounted for the largest proportion (14 studies, 70%). Within this domain, four studies addressed preterm birth [25,28-30], three addressed hypertensive disorders of pregnancy, SMM, and preeclampsia [31-33], three addressed GDM [34-36], one addressed high-risk pregnancy [37], one addressed low birth weight [38], and two addressed maternal mental health [39,40].

#### Predicting the risk of pregnancy complications

##### 1) Preterm birth prediction

Preterm birth prediction studies used either ML models based on large cohorts and administrative data or models that formalized clinical expert knowledge. In the United States, an ML model using multivariate adaptive regression splines predicted preterm birth among Black and White pregnant women while accounting for health disparities related to chronic stress and race. The race-stratified models assessed the importance of sociodemographic and psychological variables and, after cross-validation, showed acceptable discrimination, with area under the curve (AUC) values of 0.754 and 0.765, and adequate calibration [28]. In Brazil, an ML prediction model used nationwide municipal childbirth data to estimate gestational age at delivery and the probability of preterm birth. The model demonstrated practical applicability within the national primary healthcare system by providing early warnings of high preterm birth risk and enabling healthcare professionals to use available resources for preventive intervention [29]. A Chinese study of singleton pregnancies compared ML models using clinical variables from different pregnancy stages and reported excellent performance of a CatBoost model for predicting preterm birth after 26 weeks. The study also identified strong predictors of preterm birth, supporting the use of the model within healthcare providers’ clinical workflows [30]. These three studies used large datasets to predict preterm birth risk by race, country, or pregnancy stage and demonstrated the potential application of such tools in settings with limited resources.

Another study assessed preterm birth risk by formalizing clinical expert knowledge rather than developing a data-driven model. In Greece, researchers developed an FAHP model incorporating six dimensions of preterm birth risk identified through literature review and expert consensus, drawing on the clinical knowledge of 35 midwives and obstetric experts. The model was validated using 153 clinical cases [25]. Compared with expert judgment, the resulting risk score showed significant predictive power for preterm birth, suggesting that expert knowledge may offer a viable alternative when large datasets are difficult to obtain [25].

##### 2) Prediction of hypertensive disorders of pregnancy, severe maternal morbidity, and preeclampsia

One study examined the association between hypertensive disorders of pregnancy and SMM and developed an SMM prediction model [31]. Multivariate analysis identified systolic blood pressure and total cholesterol at admission for suspected hypertensive disorders of pregnancy as independent risk factors for SMM. These findings informed a nomogram specifically for pregnant women with hypertensive disorders of pregnancy, which was reported to be useful for early assessment of disease severity and SMM risk [31]. Another study used routinely collected maternal health data to predict preeclampsia [32]. Training and validation of supervised ML models identified nulliparity, prepregnancy body mass index (BMI), previous preeclampsia, maternal age, family history of hypertension, and preexisting hypertension and diabetes as key predictors. Two prediction models performed excellently in identifying preeclampsia risk [32]. A further study compared ML algorithms to identify the best model for assessing preeclampsia risk before conception using maternal clinical history [33].

##### 3) Prediction of gestational diabetes mellitus

Three studies addressed GDM. One developed an ML model for early GDM prediction using prepregnancy BMI, first-trimester abdominal circumference, age, polycystic ovary syndrome, parity, irregular menstruation, and family history of diabetes. The model allowed straightforward screening for high GDM risk before 14 weeks of gestation without blood tests [34]. Another study developed a nomogram and web calculator to predict preterm birth in women with GDM. Both the development and validation datasets showed excellent discrimination and good calibration, and the tool was reported to be useful for estimating individual preterm birth risk in this population [35]. A third study compared seven ML algorithms using first-trimester sociodemographic and obstetric characteristics to identify the best-performing model for early GDM prediction [36].

##### 4) Prediction of high-risk pregnancy and low birth weight

An ML model classified pregnancy risk as low, moderate, or high using readily obtainable clinical measures: maternal age, systolic and diastolic blood pressure, blood glucose, body temperature, and heart rate [37]. Six algorithms were compared, including multilayer perceptron (MLP), random forest, and XGBoost. MLP performed best, with particularly high accuracy and recall for high-risk pregnancy prediction [37]. These findings suggest that early screening for high-risk pregnancies may be feasible using basic vital signs without complex diagnostic tests.

A study in Ethiopia used causal ML to predict low birth weight among women with low-risk singleton pregnancies receiving care from midwives and to quantify the effects of midwife-led pregnancy care [38]. Causal k-nearest neighbors achieved the highest predictive performance. The main identified risk factors were meconium aspiration, perinatal death, intrapartum hemorrhage, need for neonatal resuscitation, and a history of reproductive organ surgery. Causal estimation also indicated that prenatal care by midwives significantly reduced low birth weight in both individual and average treatment-effect estimates [38].

##### 5) Maternal mental health management

Studies predicting antenatal mental health outcomes also inform when and how nurses can assess pregnant women early and intervene. Antenatal depression models developed using large Chinese cohort and multicenter data incorporated nursing assessment variables, including sociodemographic characteristics, lifestyle, social support, and mental health history, to provide a risk assessment approach for clinical nursing use [39,40]. Comparisons of random forest, SVM, XGBoost, and light gradient boosting machine (LightGBM), together with SHapley Additive exPlanations (SHAP) analysis, identified and visually displayed variables indicating high depression risk. These results clarified which areas nurses should prioritize during consultations and patient education [39,40]. Both studies showed substantial potential for clinical application by supporting early identification of high antenatal depression risk and timely intervention using basic information collected during prenatal visits, such as obstetric variables, education level, and coping strategies.

#### Fetal/intrapartum monitoring

A retrospective cohort study [41] developed an ML-based clinical decision support model to predict episiotomy need in women attempting vaginal delivery of a full-term, vertex, singleton fetus. As an early warning system, the model used physiological data collected during labor to predict mediolateral episiotomy risk rapidly and accurately. It was implemented online so that nurses and midwives could enter intrapartum data in real time and receive intuitive SHAP-based feedback to support standardized clinical decisions [41]. Another study trained and evaluated ML models using intrapartum FHR signals to classify delivery mode [23]. Deep learning-based ML significantly improved the efficiency of distinguishing cesarean from vaginal deliveries, demonstrating its value in supporting intrapartum decisions. A further study combined maternal ECG waveform analysis with ML to monitor pain from uterine contractions [24]. The LightGBM model achieved high accuracy in continuous labor pain monitoring, suggesting potential use as an objective pain assessment tool in other healthcare settings.

#### Clinical decision support

An AI-based VBAC prediction system was developed using medical records of women with previous cesarean delivery and evaluated within a platform supporting shared decision-making [42]. The study compared tree-based CatBoost with logistic regression models for predicting VBAC success. Because the models predicted success effectively, the system was proposed as a tool to support decisions about delivery mode. Its integration into a shared decision-making platform also suggested potential use as a standardized decision aid for pregnant women and healthcare providers [42].

#### Education and nursing documentation

Robot-assisted digital education significantly improved health literacy in pregnant women with GDM compared with conventional video education. This finding supported its potential clinical use as an efficient educational strategy for this population [26]. A pregnancy nursing intervention study used a ChatGPT-4 prompt to generate care plans for a case involving preterm labor and GDM, proposing and validating nursing diagnoses, interventions, and outcomes [27]. Compared with nurse-written care plans, ChatGPT-4 correctly identified nursing domains, excluded unnecessary or inappropriate recommendations, and set appropriate priorities, including placing the highest-priority nursing problems first. Of the 24 labels generated, 16 correctly used standardized nursing terminology systems, including NANDA-International, Nursing Outcomes Classification, and Nursing Interventions Classification [27]. These findings suggest that ChatGPT-4 may assist nurses and midwives in preparing pregnancy care plans and support their documentation work.

## Discussion

This review examined AI applications in prenatal and intrapartum maternal health management, including patterns of use and outcomes relevant to nursing practice. The 20 studies covered four domains: risk prediction, fetal/intrapartum monitoring, clinical decision support, and patient education and nursing documentation. ML models predominated, with applications spanning prenatal through intrapartum care and increasingly supporting nurses’ clinical judgments. The following discussion compares these findings with previous research and considers their implications and limitations for nursing practice, education, research, and policy.

First, ML prediction models were most commonly used to estimate pregnancy complication risk. The 14 studies (70%) in this domain used random forest, SVM, XGBoost, CatBoost, and logistic regression to predict complications including preterm birth, GDM, preeclampsia, high-risk pregnancy, and SMM. This pattern is consistent with obstetric research using ML to predict maternal outcomes such as preterm birth, preeclampsia, GDM, cardiovascular risk, and postpartum hemorrhage [5,7,14,15,18,43-45]. Vasudevan et al. [45], for example, reviewed ML models that used electronic medical records to predict maternal mortality and morbidity, a trend also observed here. Sharifi-Heris et al. [15] identified reliance on single-center data as a limitation of ML models for preterm birth prediction. The inclusion in this review of a population-based study using national data [29] indicates that study scope and dataset size are beginning to address that limitation. Giaxi et al. [14] reported that AI research in obstetrics and midwifery concentrated on model development and performance evaluation. The inclusion here of a causal ML study quantifying the effects of midwife interventions [38] indicates that research is also extending beyond prediction to produce evidence for nursing interventions.

Studies using large datasets to predict preterm birth risk by race, country, or pregnancy stage [28-30] showed potential for use in settings with limited resources. This is consistent with research suggesting that AI may improve equity and access in prenatal health management [4,5,18,44]. The causal ML study quantifying midwifery intervention effects [38] also reflects an emerging use of AI in nursing intervention research [46-49]. These prediction models are closely relevant to nursing because they integrate obstetric history, anthropometric measures, lifestyle, and sociodemographic information routinely collected by nurses to support early identification of high-risk pregnancies and adjustment of care intensity and follow-up [4,50].

Antenatal depression models combining sociodemographic, psychosocial, and mental health history data from large cohorts [39,40] show the potential of AI to support early screening for antenatal depression, a known precursor to postpartum depression. These findings are consistent with Huang et al. [51], who evaluated and interpreted ML predictions of first-trimester depression risk among low-income women of color using electronic medical records and reported the predictive factors. They also align with studies emphasizing interpretable AI as a key contributor to clinical acceptability and patient-centered decision-making [21,52]. By helping nurses explain why an individual pregnant woman has been classified as having high depression risk, these findings may improve psychosocial assessment, patient education, and counseling [46,52]. Integrating these variables is also consistent with research suggesting that accounting for race and socioeconomic background may improve prediction accuracy and reduce model bias [52,53].

Depression and anxiety are shaped by biological factors and by social and cultural circumstances, including forms of expression, stigma, and support systems [47,51]. Predictions of antenatal depression must therefore be interpreted in the individual’s personal and cultural context, with careful consideration of ethical issues. Classifying a pregnant woman as having high mental health risk may expose her to stigma, while collecting and storing sensitive psychosocial information may compromise privacy. Bias against particular racial or socioeconomic groups may also worsen existing health inequalities.

Second, fetal/intrapartum monitoring studies developed systems to predict episiotomy need from real-time physiological changes during labor [41] and to classify cesarean and vaginal deliveries accurately using FHR signals [23]. These applications are consistent with the review by Lovers et al. [54], which suggested that AI-based FHR interpretation can reduce variation between readers and improve clinical consistency. ECG waveform analysis for labor pain monitoring [24] provided objective measures to complement subjective pain assessment, making it relevant to nursing tasks involving intrapartum monitoring and pain management. However, Dlugatch et al. [16] reported concerns that reliance on AI-based FHR decisions could weaken healthcare professionals’ skills and judgment. Improvements in technical performance should therefore be accompanied by training for nurses and midwives in clinical AI use and in the assessment, monitoring, and judgment skills needed to apply it.

Third, AI showed potential to support clinical decisions in intrapartum situations requiring immediate judgment. A study integrating AI prediction with a shared decision-making platform [42] reported benefits in providing evidence-based information while respecting pregnant women’s autonomy. This example combines AI with the maternal nursing advocacy role described by Penner et al. [43]. Integration into practice nevertheless requires caution because labor involves substantial physiological variability and individual differences, and outcomes are strongly influenced by factors that are difficult to quantify, including emotional state, preferences, and cultural context. Further validation and safety standards in clinical settings are needed before such integration [55-58].

Fourth, AI applications in patient education and nursing documentation extended beyond prediction to direct nursing interventions and practical assistance. Robot-assisted digital education significantly improved maternal health literacy among women with GDM [26]. This finding is consistent with studies suggesting that digital education, through personalized content and repeated learning, can strengthen maternal self-management [45]. O’Connor et al. [55] reported that nursing and midwifery AI research concentrated on clinical decision support and diagnostic assistance, with documentation research still at an early stage. A study of ChatGPT-4 care plan documentation [27], however, generated nursing diagnoses, interventions, and outcomes using standardized nursing terminology accurately. This provides empirical support for using an LLM to prepare care plans consistent with standardized terminology and reduce documentation burden. AI-based education may nevertheless worsen inequalities for people with limited digital literacy or technology access [44,50]. Adoption should therefore be tailored to the population and setting and accompanied by an appropriate balance of face-to-face education.

Effective use of AI in maternal nursing requires technical performance, nurses’ competencies, and suitable conditions for clinical implementation. AI literacy includes understanding algorithm principles and performance measures, such as AUC, sensitivity, and specificity, and recognizing model limitations and potential biases [56,58]. Nurses also need clinical interpretation skills to combine AI-generated risk estimates with each pregnant woman’s clinical, social, and cultural circumstances when planning care [55]. Communication skills are needed to explain interpretable AI outputs, such as SHAP analyses and nomograms, in understandable language and respond sensitively to women’s emotions [22,43]. Implementation should respect maternal autonomy, equitable healthcare access, and privacy. AI outputs should inform decisions alongside nurses’ professional judgment and emotional support, without becoming the sole basis for decisions [28,38]. Under these conditions, AI can help nurses provide more precise, individualized, person-centered care.

Nursing education must prepare nurses to use AI safely and effectively in maternal health management. Curricula should systematically address AI literacy, interpretation of explainable AI, algorithmic bias, and ethical issues [48,56]. In clinical settings, simulations based on actual obstetric scenarios should develop nurses’ ability to incorporate AI predictions into counseling and shared decisions with pregnant women. Nurses should also participate actively in AI development, validation, implementation, and evaluation [55,58] so that the technology reflects clinical needs.

Policy should establish the institutional conditions needed to integrate AI into national maternal health management systems. Priorities include standardizing clinical data and ensuring interoperability, setting licensing and safety verification standards for AI-based medical devices, and establishing oversight of algorithmic bias and equity. Policymaking should incorporate nurses’ clinical perspectives and those of other healthcare professionals to ensure that AI policies address the practical needs of healthcare users and practitioners [4,5,18,44].

By systematically examining AI use and its nursing implications in prenatal and intrapartum maternal health management, this review described links to nursing practice across risk prediction, fetal/intrapartum monitoring, clinical decision support, and patient education and nursing documentation. Clinical application of these findings requires continued assessment of AI effectiveness, equity, and ethical implications across nursing practice, education, research, and policy, alongside development of strategies for integration into nursing care. These efforts can support person-centered, equitable, and safe use of AI in maternal health management.

This review has several limitations. Most studies used retrospective cohort designs and were concentrated in particular regions, limiting generalizability. Few directly examined nurses’ experiences of using AI or the process of clinical integration. Critical appraisal was not undertaken as part of this scoping review, and the included studies used diverse designs with differing levels of evidence. The findings should therefore be interpreted cautiously in light of individual studies’ methodological quality.

Future systematic reviews should assess evidence quality for specific AI technologies or clinical application domains. Further work should include multicenter studies across diverse cultural and regional settings, intervention studies evaluating AI tools’ clinical utility and effects on nursing outcomes, and qualitative studies examining nurses’ experiences and competency needs.

## Figures and Tables

**Figure 1.**
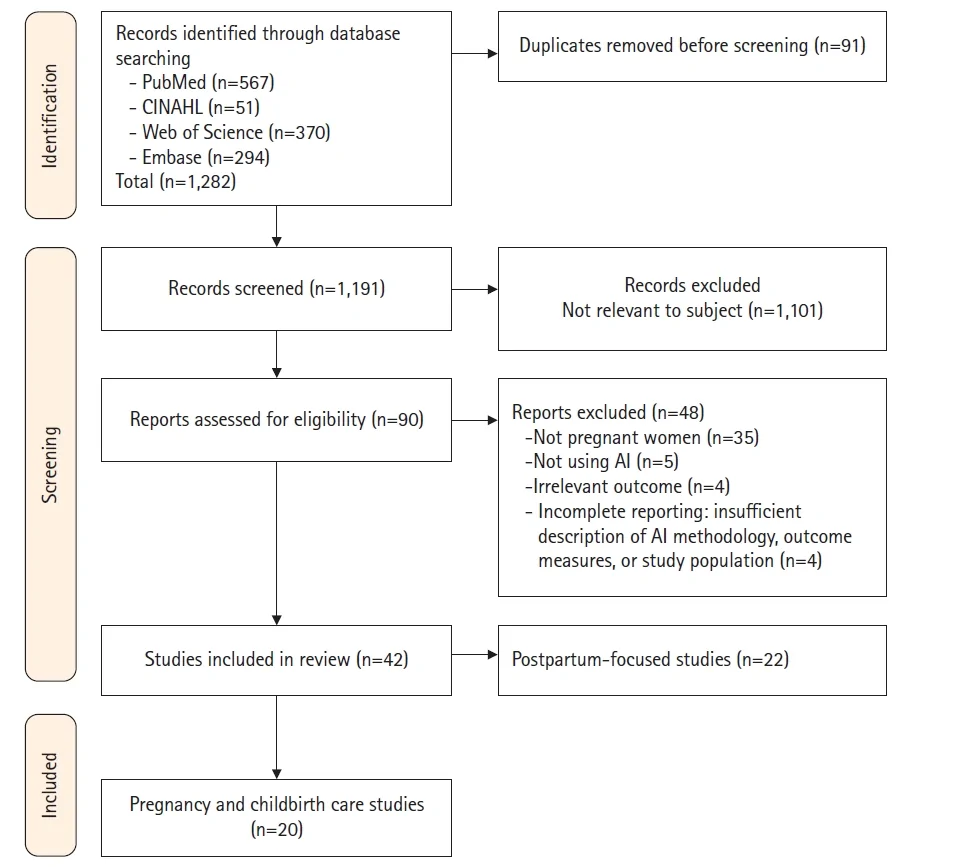
PRISMA-ScR (Preferred Reporting Items for Systematic Reviews and Meta-Analyses extension for Scoping Reviews) flow diagram of study selection. AI: Artificial intelligence.

**Table 1.** Characteristics of the included studies (N=20)

Characteristic	Category	n (%)
Publication year	2017	1 (5.0)
	2021	2 (10.0)
	2022	1 (5.0)
	2024	9 (45.0)
	2025	7 (35.0)
Country	China	7 (35.0)
	Taiwan	3 (15.0)
	Türkiye	2 (10.0)
	United States	2 (10.0)
	Brazil	1 (5.0)
	Ethiopia	1 (5.0)
	United Kingdom	1 (5.0)
	Greece	1 (5.0)
	Australia	1 (5.0)
	Multi-country	1 (5.0)
Study design	Retrospective cohort study	14 (70.0)
	Retrospective signal analysis	2 (10.0)
	Population-based study	1 (5.0)
	Model development and validation	1 (5.0)
	Randomized controlled trial	1 (5.0)
	Quality improvement study	1 (5.0)
AI technology type	ML-based prediction model	15 (75.0)
	ML with physiological signal analysis	2 (10.0)
	Fuzzy analytic hierarchy process	1 (5.0)
	Robot-assisted education system	1 (5.0)
	Large language model (ChatGPT-4)	1 (5.0)
Application domain	Risk prediction	14 (70.0)
	Fetal/intrapartum monitoring	3 (15.0)
	Clinical decision support	1 (5.0)
	Patient education	1 (5.0)
	Nursing documentation	1 (5.0)
Pregnancy stage	Prenatal, general	13 (65.0)
	First trimester	2 (10.0)
	Mid-to-late pregnancy	1 (5.0)
	Prenatal-intrapartum	1 (5.0)
	Intrapartum	3 (15.0)
Target health outcome	Preterm birth	4 (20.0)
	Gestational diabetes mellitus	3 (15.0)
	Antenatal/perinatal depression	2 (10.0)
	Preeclampsia	2 (10.0)
	High-risk pregnancy, general	1 (5.0)
	Severe maternal morbidity	1 (5.0)
	Episiotomy	1 (5.0)
	Delivery mode/VBAC	1 (5.0)
	Cesarean delivery classification	1 (5.0)
	Labor pain assessment	1 (5.0)
	Low birth weight	1 (5.0)
	Maternal health literacy	1 (5.0)
	Nursing care plan documentation	1 (5.0)

AI: Artificial intelligence; ML: machine learning; VBAC: vaginal birth after cesarean delivery.

**Table 2.** Details of the included studies (N=20)

Author (year)	Country	Study design	Sample size	Pregnancy stage	AI technology type	Application domain	Key findings
Risk prediction models (n=14)							
Barbounaki and Sarantaki [25] (2022)	Greece	Model development and validation	35 experts; 153 validation cases	Prenatal	Fuzzy inference system (FAHP)	Risk prediction for preterm birth	An FAHP model was developed to assess preterm birth risk; predictive validity was confirmed against expert judgment (*p*<0.05)
Kim et al. [28] (2024)	United States	Retrospective cohort	359,855 (weighted sample)	Prenatal	ML (MARS; race/ethnicity-specific models)	Risk prediction for preterm birth	An interpretable, race-specific model used chronic stress exposures to predict preterm birth and account for racial health disparities (AUC, 0.754–0.765)
Rocha et al. [29] (2021)	Brazil	Population-based study	3,876,666	Prenatal	ML (random forest and other algorithms)	Risk prediction for preterm birth	A preterm birth risk stratification model used national data from 3,929 municipalities and demonstrated applicability in settings with limited resources
Yu et al. [30] (2024)	China/United Kingdom	Retrospective cohort	355,062	Prenatal	ML (comparison of multiple algorithms)	Risk prediction for preterm birth	ML algorithms were compared for predicting preterm birth in singleton pregnancies, and the best-performing algorithm was identified
Bian et al. [31] (2024)	China	Retrospective cohort	970	Prenatal	ML (multiple algorithms)	Risk prediction for severe maternal morbidity	A model was developed to predict severe morbidity in women with hypertensive disorders of pregnancy; predictive performance was reported
Tiruneh et al. [32] (2024)	Australia	Retrospective cohort	48,250	Prenatal	ML (routinely collected clinical data)	Risk prediction for preeclampsia	An ML model used routinely collected clinical data to predict preeclampsia and demonstrated feasibility for practical implementation
Kaya et al. [33] (2024)	Türkiye	Retrospective cohort	100	Prenatal	ML (multiple algorithms)	Risk prediction for preeclampsia	Algorithms were compared for assessing preeclampsia risk before conception using maternal clinical history
Wang et al. [34] (2021)	China	Retrospective cohort	1,139	First trimester	ML (random forest, logistic regression)	Risk prediction for GDM	A first-trimester GDM prediction model required no blood tests and demonstrated applicability in primary healthcare
Li et al. [35] (2024)	China	Retrospective cohort	1,879	Prenatal	ML (multiple algorithms)	Preterm birth risk prediction in women with GDM	A preterm birth prediction model was developed and validated in women with GDM; AUC values were reported
Kaya et al. [36] (2024)	Türkiye	Retrospective cohort	97	First trimester	ML (comparison of 7 algorithms)	Risk prediction for GDM	Performance of 7 ML algorithms was compared for early GDM prediction using first-trimester sociodemographic and obstetric characteristics
Pi et al. [37] (2025)	China	Retrospective cohort	1,014	Prenatal	ML (multiple algorithms)	Risk prediction for high-risk pregnancy	An ML model was developed to predict high-risk pregnancy; AUC values were reported
Moges et al. [38] (2025)	Ethiopia	Retrospective cohort	1,166	Prenatal	Causal ML	Risk prediction for low birth weight	A causal ML model predicted low birth weight in a midwife-led continuity care intervention
Hu et al. [39] (2025)	China	Retrospective cohort	20,950	Prenatal	ML (multiple algorithms)	Risk prediction for antenatal depression	An antenatal depression prediction model was developed and validated, and key risk factors were identified for clinical application
Wang et al. [40] (2025)	China	Retrospective cohort with external validation	499	Mid-to-late pregnancy	Interpretable ML	Risk prediction for perinatal depression	An interpretable ML model predicted perinatal depression in mid-to-late pregnancy and was externally validated in Chinese women
Fetal/intrapartum monitoring (n=3)							
Fergus et al. [23] (2017)	United Kingdom	Retrospective signal analysis	598	Intrapartum	ML (SVM, ANN, MLP, etc.)	Fetal monitoring (cesarean delivery classification)	ML algorithms classified cesarean versus vaginal delivery using FHR signals; performance was compared across models
Chu et al. [24] (2024)	Taiwan	Retrospective signal analysis	6,010	Intrapartum	ML with ECG waveform analysis	Intrapartum monitoring (labor pain assessment)	ML and ECG waveform analysis were used to monitor pain from uterine contractions; pain classification accuracy was reported
Hu et al. [41] (2025)	China	Retrospective cohort	1,191	Intrapartum	ML (SVM, RF, LightGBM, XGBoost, LR, KNN)	Intrapartum monitoring (episiotomy prediction)	An AI model predicted mediolateral episiotomy risk through an online early warning system; SVM performed best (AUC=0.882, recall=0.981)
Clinical decision support (n=1)							
Yang et al. [42] (2024)	Taiwan	Retrospective cohort	40	Prenatal-intrapartum	AI-based prediction system (ML)	Clinical decision support (VBAC prediction)	An AI-based VBAC prediction system was integrated into a shared decision-making platform, and outcomes of delivery-mode decision support were evaluated
Education and nursing documentation (n=2)							
Yang et al. [26] (2025)	Taiwan	Randomized controlled trial (RCT)	66	Prenatal	Robot-assisted hybrid system	Patient education (health literacy)	Robot-assisted digital education significantly improved maternal health literacy in an RCT, with significant between-group differences
Johnson et al. [27] (2025)	United States	Descriptive study (quality improvement)	22 (exemplar cases)	Prenatal	ChatGPT-4 (large language model)	Nursing documentation (nursing care plans)	Perinatal nursing care plans generated by ChatGPT-4 were comparable to nurse-written plans, demonstrating potential to improve plan quality and reduce documentation burden

AI: Artificial intelligence; ANN: artificial neural network; AUC: area under the curve; ECG: electrocardiography; FAHP: fuzzy analytic hierarchy process; FHR: fetal heart rate; GDM: gestational diabetes mellitus; KNN: k-nearest neighbors; LightGBM: Light Gradient Boosting Machine; LR: logistic regression; MARS: multivariate adaptive regression splines; ML: machine learning; MLP: multilayer perceptron; RCT: randomized controlled trial; RF: random forest; SVM: support vector machine; VBAC: vaginal birth after cesarean; XGBoost: Extreme Gradient Boosting.

## Supplementary materials

Further details on supplementary materials are presented online (available at https://doi.org/10.4069/whn.2026.09.04).

Supplementary Material 1. Search strategies

Supplementary Material 2. Characteristics and key findings of studies included in the scoping review (N=20)
