# Supplementary material for: Artificial intelligence technologies in pregnancy and childbirth care: a scoping review from a nursing perspective
Source: Womens Health Nurs. 2026 Sep 29;32(3):190–204. doi: 10.4069/whn.2026.09.04 (PMC13654686; doi:10.4069/whn.2026.09.04)
Supplement: Supplementary file 1 [file whn-2026-09-04-Supplementary-Material-1.pdf]

Supplementary Material 1. Search strategies

Database	Search terms
PubMed/MEDLINE	("pregnancy"[Text Word] OR "Fetus"[Text Word] OR "Infant"[Text Word] OR "Newborn"[Text Word] OR "baby"[Text Word] OR "pregnant"[Text Word] OR "maternal"[Text Word] OR "preterm"[Text Word] OR "fetal"[Text Word] OR "birth"[Text Word] OR "labor"[Text Word] OR "perinatal"[Text Word] OR "prenatal"[Text Word] OR "antenatal"[Text Word] OR "gestational"[Text Word] OR "gestation"[Text Word] OR "Obstetrics"[Text Word] OR "Gynecology"[Text Word] OR "pregnancy"[MeSH Major Topic] OR "pregnancy outcome"[MeSH Major Topic] OR "Fetus"[MeSH Major Topic] OR "Infant"[MeSH Major Topic] OR "infant, newborn"[MeSH Major Topic] OR "neonatal screening"[MeSH Major Topic] OR "prenatal diagnosis"[MeSH Major Topic] OR "prenatal care"[MeSH Major Topic] OR "premature birth"[MeSH Major Topic] OR "infant, premature"[MeSH Major Topic] OR "obstetric labor, premature"[MeSH Major Topic] OR "infant, extremely premature"[MeSH Major Topic] OR "infant, extremely low birth weight"[MeSH Major Topic] OR "Gynecology"[MeSH Major Topic] OR "Obstetrics"[MeSH Major Topic] OR "fetal monitoring"[MeSH Major Topic]) AND (("artificial intelligence"[Text Word] OR "machine learning"[Text Word] OR "predictive model"[Text Word] OR "neural network"[Text Word] OR "deep learning"[Text Word] OR "natural language processing"[Text Word] OR "NLP"[Text Word] OR "fuzzy logic"[Text Word] OR "ontology"[Text Word] OR "knowledge graph"[Text Word] OR "computer heuristics"[MeSH Major Topic] OR "machine learning"[MeSH Major Topic] OR "natural language processing"[MeSH Major Topic] OR "biological ontologies"[MeSH Major Topic] OR "neural networks, computer"[MeSH Major Topic] OR "robotics"[MeSH Major Topic] OR "deep learning"[MeSH Major Topic] OR (("artifical"[All Fields] OR "artifically"[All Fields]) AND "intelligence"[MeSH Major Topic])) NOT ("dataset"[Publication Type] OR "review"[Publication Type] OR "systematic review"[Publication Type] OR "meta analysis"[Publication Type] OR "case reports"[Publication Type] OR "comment"[Publication Type] OR "editorial"[Publication Type] OR "news"[Publication Type] OR "English abstract"[Publication Type] OR "congress"[Publication Type] OR "overall"[Publication Type])) AND ("nurs*"[All Fields] OR "nursing"[All Fields] OR "nursing care"[All Fields] OR "nursing practice"[All Fields] OR "maternal nursing"[All Fields] OR "obstetric nursing"[All Fields] OR "midwi*"[All Fields] OR "midwifery"[All Fields] OR "midwife"[All Fields] OR "midwives"[All Fields] OR "midwifery care"[All Fields])
	Pregnancy[MeSH Major Topic]: "pregnancy"[MeSH Major Topic]<br>Pregnancy Outcome[MeSH Major Topic]: "pregnancy outcome"[MeSH Major Topic]<br>Fetus[MeSH Major Topic]: "fetus"[MeSH Major Topic]<br>Infant[MeSH Major Topic]: "infant"[MeSH Major Topic]<br>Infant, Newborn[MeSH Major Topic]: "infant, newborn"[MeSH Major Topic]<br>Neonatal Screening[MeSH Major Topic]: "neonatal screening"[MeSH Major Topic]<br>Prenatal Diagnosis[MeSH Major Topic]: "prenatal diagnosis"[MeSH Major Topic]<br>Depression, Postpartum[MeSH Major Topic]: "depression, postpartum"[MeSH Major Topic]<br>Prenatal Care[MeSH Major Topic]: "prenatal care"[MeSH Major Topic]<br>Premature Birth[MeSH Major Topic]: "premature birth"[MeSH Major Topic]<br>Infant, Premature[MeSH Major Topic]: "infant, premature"[MeSH Major Topic]<br>Obstetric Labor, Premature[MeSH Major Topic]: "obstetric labor, premature"[MeSH Major Topic]<br>Infant, Extremely Premature[MeSH Major Topic]: "infant, extremely premature"[MeSH Major Topic]<br>Infant, Extremely Low Birth Weight[MeSH Major Topic]: "infant, extremely low birth weight"[MeSH Major Topic]<br>Gynecology[MeSH Major Topic]: "gynecology"[MeSH Major Topic]<br>Obstetrics[MeSH Major Topic]: "obstetrics"[MeSH Major Topic]<br>Fetal Monitoring[MeSH Major Topic]: "fetal monitoring"[MeSH Major Topic]<br>Computer Heuristics[MeSH Major Topic]: "computer heuristics"[MeSH Major Topic]<br>Machine Learning[MeSH Major Topic]: "machine learning"[MeSH Major Topic]<br>Natural Language Processing[MeSH Major Topic]: "natural language processing"[MeSH Major Topic]<br>Biological Ontologies[MeSH Major Topic]: "biological ontologies"[MeSH Major Topic]<br>Neural Networks, Computer[MeSH Major Topic]: "neural networks, computer"[MeSH Major Topic]<br>Robotics[MeSH Major Topic]: "robotics"[MeSH Major Topic]<br>Deep Learning[MeSH Major Topic]: "deep learning"[MeSH Major Topic]<br>Artifical: "artifical"[All Fields] OR "artifically"[All Fields]<br>Intelligence[MeSH Major Topic]: "intelligence"[MeSH Major Topic]
Web of Science	TS = (pregnancy OR pregnant OR fetus OR fetal OR baby OR newborn OR infant OR maternal OR birth OR labor OR delivery OR preterm OR gestational OR gestation OR postpartum OR perinatal OR prenatal OR antenatal OR obstetrics OR gynecology OR "pregnancy outcome" OR "prenatal care" OR "neonatal screening" OR "prenatal diagnosis" OR "premature birth" OR "infant, premature" OR "extremely premature infant" OR "extremely low birth weight infant" OR "obstetric labor, premature" OR "fetal monitoring") AND TS = ("artificial intelligence" OR "machine learning" OR "predictive model" OR "neural network" OR "deep learning" OR "natural language processing" OR "NLP" OR "fuzzy logic" OR "ontology" OR "knowledge graph" OR "computer heuristics" OR "biological ontologies" OR "robotics" OR "chatbot") and Review Article or Meeting Abstract or Early Access or Article (Document Types) and Proceeding Paper or Early Access or Meeting Abstract (Exclude – Document Types) and Book Chapters or Publication With Expression Of Concern or Withdrawn Publication or Review Article or Retracted Publication (Exclude – Document Types) AND TS = (nurs* OR "nursing" OR "nursing care" OR "nursing practice" OR "maternal nursing" OR "obstetric nursing" OR midwi* OR "midwifery" OR "midwife" OR "midwives" OR "midwifery care")

(Continued to the next page)

Supplementary Material 1. Continued

Database	Search terms
CINHAL	(MH "Pregnancy+" OR MH "Pregnant Women+" OR MH "Fetus+" OR MH "Infant+" OR MH "Infant, Newborn+" OR MH "Maternal Health+" OR MH "Labor, Obstetric+" OR MH "Prenatal Care+" OR MH "Postpartum Period+" OR MH "Obstetrics+" OR MH "Gynecology+" OR MH "Perinatal Care+" OR MH "Premature Birth+" OR MH "Infant, Premature+" OR MH "Infant, Extremely Premature+" OR MH "Infant, Extremely Low Birth Weight+" OR MH "Prenatal Diagnosis+" OR MH "Neonatal Screening+" OR MH "Fetal Monitoring+" OR TI pregnancy OR TI fetus OR TI infant OR TI newborn OR TI baby OR TI pregnant OR TI maternal OR TI preterm OR TI fetal OR TI birth OR TI labor OR TI perinatal OR TI prenatal OR TI antenatal OR TI gestational OR TI gestation OR TI postpartum OR TI obstetrics OR TI gynecology OR AB pregnancy OR AB fetus OR AB infant OR AB newborn OR AB baby OR AB pregnant OR AB maternal OR AB preterm OR AB fetal OR AB birth OR AB labor OR AB perinatal OR AB prenatal OR AB antenatal OR AB gestational OR AB gestation OR AB postpartum OR AB obstetrics OR AB gynecology) AND (MH "Artificial Intelligence+" OR MH "Machine Learning+" OR MH "Natural Language Processing+" OR MH "Robotics+" OR MH "Neural Networks+" OR MH "Ontologies+" OR TI "artificial intelligence" OR TI "machine learning" OR TI "predictive model" OR TI "neural network" OR TI "deep learning" OR TI "natural language processing" OR TI "fuzzy logic" OR TI "ontology" OR TI "knowledge graph" OR TI "NLP" OR TI "chatbot" OR TI "conversational agent" OR AB "artificial intelligence" OR AB "machine learning" OR AB "predictive model" OR AB "neural network" OR AB "deep learning" OR AB "natural language processing" OR AB "fuzzy logic" OR AB "ontology" OR AB "knowledge graph" OR AB "NLP" OR AB "chatbot" OR AB "conversational agent") AND (MH "Nursing+" OR MH "Nursing Care+" OR MH "Nursing Practice+" OR MH "Maternal Nursing+" OR MH "Obstetric Nursing+" OR MH "Midwifery+" OR MH "Midwives+" OR TI "nurs*" OR TI "nursing care" OR TI "nursing practice" OR TI "maternal nursing" OR TI "obstetric nursing" OR TI "midwi*" OR TI "midwifery" OR TI "midwife" OR TI "midwives" OR AB "nurs*" OR AB "nursing care" OR AB "nursing practice" OR AB "maternal nursing" OR AB "obstetric nursing" OR AB "midwi*" OR AB "midwifery" OR AB "midwife" OR AB "midwives")
Embase	('pregnancy'/exp OR 'pregnant woman'/exp OR 'maternal health'/exp OR 'fetus'/exp OR 'newborn'/exp OR 'infant'/exp OR 'perinatal period'/exp OR 'prenatal care'/exp OR 'postpartum period'/exp OR 'obstetrics'/exp OR 'gynecology'/exp OR pregnancy:ti,ab,kw OR pregnant:ti,ab,kw OR fetus:ti,ab,kw OR newborn:ti,ab,kw OR infant:ti,ab,kw OR maternal:ti,ab,kw OR gestational:ti,ab,kw OR antenatal:ti,ab,kw OR prenatal:ti,ab,kw OR labor:ti,ab,kw OR birth:ti,ab,kw) AND 'artificial intelligence'/exp OR 'machine learning'/exp OR 'deep learning'/exp OR 'natural language processing'/exp OR 'predictive model':ti,ab,kw OR 'neural network':ti,ab,kw OR 'chatbot':ti,ab,kw OR 'knowledge graph':ti,ab,kw OR 'ontology':ti,ab,kw OR 'fuzzy logic':ti,ab,kw OR 'robotics'/exp OR 'artificial intelligence':ti,ab,kw OR 'machine learning':ti,ab,kw OR 'deep learning':ti,ab,kw OR 'natural language processing':ti,ab,kw OR 'NLP':ti,ab,kw OR 'chatbot':ti,ab,kw) AND ('nursing'/exp OR 'nurse'/exp OR 'nursing care'/exp OR 'midwife'/exp OR 'nursing':ti,ab,kw OR 'nursing care':ti,ab,kw OR 'maternal nursing':ti,ab,kw OR 'obstetric nursing':ti,ab,kw OR 'nursing practice':ti,ab,kw OR 'midwife':ti,ab,kw OR 'midwives':ti,ab,kw OR 'midwifery':ti,ab,kw OR 'midwifery care':ti,ab,kw) NOT ('review'/it OR 'meta analysis'/it OR 'systematic review'/it OR 'case report'/it OR 'editorial'/it OR 'letter'/it OR 'conference paper'/it)

https://doi.org/10.4069/whn.2026.09.04
