# Supplementary material for: Artificial intelligence technologies in pregnancy and childbirth care: a scoping review from a nursing perspective
Source: Womens Health Nurs. 2026 Sep 29;32(3):190–204. doi: 10.4069/whn.2026.09.04 (PMC13654686; doi:10.4069/whn.2026.09.04)
Supplement: Supplementary file 2 [file whn-2026-09-04-Supplementary-Material-2.pdf]

Supplementary Material 2. Characteristics and key findings of studies included in the scoping review (N=20)

Author (year)	Research aim (keywords)	Participants (details)	AI application (details)	Nursing relevance
Fergus et al. [23] (2017)	FHR signal-based classification of cesarean vs. vaginal delivery	UK; n = 598; Retrospective signal analysis; Intrapartum	SVM, ANN, MLP, etc.; FHR signal-based; Cesarean vs. vaginal delivery classification	NA
Chu et al. [24] (2024)	ECG waveform-based labor pain monitoring	Taiwan; n = 6,010; Retrospective signal analysis; Intrapartum	ML+ECG waveform analysis; Uterine contraction-induced pain; Pain classification accuracy reported	NA
Barbounaki and Sarantaki [25] (2022)	Fuzzy inference-based preterm birth risk assessment	Greece; n = 35 experts+153 validation cases; Model development	Fuzzy inference system; FAHP; Expert knowledge-based risk scoring	Midwifery faculty co-authors (University of West Attica); Midwives integrating clinical intuition into AI-assisted decision-making explicitly stated
Yang et al. [26] (2025)	Robot-assisted digital education for health literacy in GDM	Taiwan; n = 66 GDM women; RCT; Prenatal	Robot-assisted digital education; Personalized content delivery; Comparison with video education	Directly designed as a nursing education intervention (RCT); Significant improvement in health literacy reported in paper
Johnson et al. [27] (2025)	ChatGPT-4-generated perinatal nursing care plans	Clinical cases: preterm labor+GDM (NA)	ChatGPT-4; Nursing care plan prompts; NANDA-I, NOC, NIC standardized terminology validation	All authors from nursing faculty (University of Florida, Columbia University); Adherence to standardized nursing terminology confirmed; Potential to reduce documentation burden explicitly stated
Kim et al. [28] (2024)	Race-specific preterm birth risk prediction	USA; n = 359,855 (weighted sample); Retrospective cohort; Prenatal	MARS; Race/ethnicity-specific models; AUC, 0.754–0.765	Nursing faculty co-author (Emory University School of Nursing); Chronic stress and racial disparities explicitly addressed
Rocha et al. [29] (2021)	Population-based preterm birth risk stratification	Brazil; n = 3,876,666; Population-based study; Prenatal	Random Forest & other ML; Administrative & sociodemographic variables; 3,929 municipalities	NA
Yu et al. [30] (2024)	Comparison of ML algorithms for preterm birth prediction in singleton pregnancies	China/UK; n = 355,062; Retrospective cohort; Prenatal	Multiple ML algorithms; Algorithm comparison; Clinical knowledge-based variable selection	NA
Bian et al. [31] (2024)	SMM prediction in hypertensive disorders	China; n = 970; Retrospective cohort; Prenatal	Multiple ML algorithms; Nomogram; SBP & total cholesterol as key predictors	NA
Tiruneh et al. [32] (2024)	Preeclampsia prediction using routinely collected clinical data	Australia; n = 48,250; Retrospective cohort; Prenatal	ML (routinely collected clinical data); Supervised learning; Key predictor identification	Emphasis on use of routinely collected data; Practical implementation feasibility explicitly discussed
Kaya et al. [33] (2024a)	Preeclampsia risk pre-assessment using preconception history	Türkiye; n = 100; Retrospective cohort; Preconception	Multiple ML algorithms; Preconception clinical history; Algorithm performance comparison	NA
Wang et al. [34] (2021)	Early prediction of GDM	China; n = 1,139; Retrospective cohort; First trimester	Random Forest, Logistic Regression; Non-invasive variables (BMI, abdominal circumference, age, PCOS, etc.)	Nursing faculty co-author; Developed as a model applicable in primary care settings; All variables obtainable during routine antenatal visits (stated in paper)
Li et al. [35] (2024)	Preterm birth risk nomogram in women with GDM	China; n = 1,879; Retrospective cohort; Prenatal	Multiple ML algorithms; Stepwise logistic regression; Nomogram & web calculator	NA
Kaya et al. [36] (2024b)	Early GDM prediction in first trimester	Türkiye; n = 97; Retrospective cohort; First trimester	7 ML algorithms compared; Sociodemographic & obstetric characteristics; Performance comparison	NA

(Continued to the next page)

https://doi.org/10.4069/whn.2026.09.04

Supplementary Material 2. Continued

Author (year)	Research aim (keywords)	Participants (details)	AI application (details)	Nursing relevance
Pi et al. [37] (2025)	High-risk pregnancy classification model	China; n = 1,014; Retrospective cohort; Prenatal	Multiple ML algorithms; AUC performance metrics; EHR-based routine data	Paper explicitly states AI can compensate for junior nurses' limited clinical decision-making competency
Moges et al. [38] (2025)	Low birth weight prediction; Quantifying effect of midwife-led care	Ethiopia; n = 1,166; Retrospective cohort; Prenatal	Causal ML; CKNN; Causal inference analysis	Applied to midwife-led continuity of care intervention; Individual and average treatment effects of midwife care quantified (stated in paper)
Hu et al. [39] (2025)	Antenatal depression risk prediction	China; n = 20,950; Retrospective cohort; Prenatal	Multiple ML algorithms; SHAP analysis; Sociodemographic & psychosocial variables	NA
Wang et al. [40] (2025)	Interpretable ML model for perinatal depression prediction	China; n = 499; Retrospective cohort+external validation; Mid-to-late pregnancy	Interpretable ML; SHAP analysis; Externally validated	NA
Hu et al. [41] (2025)	Episiotomy risk prediction & online early warning system during labor	China; n = 1,191; Retrospective cohort; Intrapartum	SVM, RF, LightGBM, XGBoost, LR, KNN; SHAP-based online early warning system (AUC, 0.882; recall, 0.981)	Nurses and midwives input real-time data during labor and receive SHAP-based feedback; Standardized clinical decision support explicitly stated
Yang et al. [42] (2024)	VBAC success prediction+SDM platform integration	Taiwan; n = 40; Retrospective cohort; Prenatal–intrapartum	CatBoost, Logistic regression; AI-based prediction system; Integrated into SDM platform	AI-based VBAC prediction integrated into SDM platform; Support for birth mode decision explicitly stated

AI: Artificial intelligence; ANN: artificial neural network; AUC: area under the curve; BMI: body mass index; CKNN: causal k-nearest neighbors; ECG: electrocardiogram; EHR: electronic health record; FAHP: fuzzy analytic hierarchy process; FHR: fetal heart rate; GDM: gestational diabetes mellitus; KNN: k-nearest neighbors; LightGBM: Light Gradient Boosting Machine; LR: logistic regression; MARS: multivariate adaptive regression splines; ML: machine learning; MLP: multilayer perceptron; NA: not applicable; NANDA-I: NANDA International; NIC: Nursing Interventions Classification; NOC: Nursing Outcomes Classification; PCOS: polycystic ovary syndrome; RCT: randomized controlled trial; RF: random forest; SBP: systolic blood pressure; SDM: shared decision-making; SHAP: SHapley Additive exPlanations; SMM: severe maternal morbidity; SVM: support vector machine; VBAC: vaginal birth after cesarean; XGBoost: eXtreme Gradient Boosting.

https://doi.org/10.4069/whn.2026.09.04
